# Microplastics in Seabird Feces from Coastal Areas of Central Chile

**DOI:** 10.3390/ani13182840

**Published:** 2023-09-07

**Authors:** Sebastian Mendez-Sanhueza, Mariett Torres, Karla Pozo, Gabriela Del Aguila, Fabián Hernandez, Camila Jacobsen, Diana Echeverry

**Affiliations:** 1 Escuela de Medicina Veterinaria, Facultad Ciencias de la Naturaleza, Universidad San Sebastián, Concepción 4081339, Chile; smendezs2@correo.uss.cl (S.M.-S.); gabydelagui@gmail.com (G.D.A.);; 2 Facultad de Ingeniería, Arquitectura y Diseño, Universidad San Sebastián, Concepción 4081339, Chile

**Keywords:** gulls, microplastic, plastic, penguins, pollution, *Spheniscus*

## Abstract

**Simple Summary:**

Wildlife species are sentinels that indicate the state of health of ecosystems. One of the main current problems is plastic contamination of aquatic and terrestrial environments, directly or indirectly affecting wildlife and humans by introducing plastic particles into the food chain. In animal feces, it is possible to identify the presence of microplastics as indicators of environmental plastic pollution. This study aimed to determine if there was evidence of the circulation of microplastic in a group of seabirds that entered a wildlife rehabilitation center in Chile. The results indicated the presence of microfibers, giving evidence of plastic pollution. The visibility of the problem will allow the establishment of measures for reducing plastic pollution and surveillance of the marine ecosystem, including the interactions among the different species that comprise it.

**Abstract:**

Pollution from plastic waste thrown into the ocean affects all levels of the food chain. Marine species of birds are affected by plastic particles of different sizes, especially the mesoplastics (1 to 10 mm) found in their digestive tract, which mainly cause obstructions. In the case of microplastics (1.000 µm to 1 mm), their presence in the digestive tract of these species has been widely reported. We studied fecal samples of the Dominican gull (*Larus dominicanus*) (*n* = 14), Magellanic penguins (*Spheniscus magellanicus*) (*n* = 8), and Humboldt penguin (*Spheniscus humboldti*) (*n* = 1) obtained from the Wildlife Rehabilitation Center of the Biobío region, Chile. Microfibers of various colors were present in the feces of Dominican gulls and Magellanic penguins, corresponding mainly in composition to polypropylene (PP) (83%) and rayon (77%). These results demonstrate that microplastic particles occur in the coastal environments of central Chile and suggest that they are probably circulating in the food chain.

## 1. Introduction

Plastic has been one of the main materials used in the last century thanks to its durability and low price [1]. However, the practice of discarding this material has shown exponential growth over the years, with the negative impacts from its accumulation identified since the 1950s [2]. The amount of plastic produced is overwhelming: around 400 million tons of plastic waste have been generated from 1950 to 2021, approximately 150 million tons have ended up in the oceans, and about 12 million tons of plastic end up in the ocean as waste [3,4]. Among this plastic waste, we find small particles, which are called “microplastics” (1.000 µm to 1 mm) [5,6,7]. These microplastics are divided into primary microplastics, manufactured to be microscopic, typically used in facial cleansers and cosmetics. However, secondary microplastics are described as small plastic pieces derived from other larger plastic compounds [8].

The study of the appearance of smaller microplastics, such as microfibers (<1 mm), has become necessary because these fibers are composed of natural materials chemically modified by the textile industry. For example, wood fibers can be modified for reinforcement using plastic polymers. Microplastics have become part of the trophic chain of animals because some animals, such as fish, consume these fibers by mistaking them for food [9]. In addition, contact and exposure of birds to marine surface waters contaminated with microplastics are becoming increasingly frequent [10,11].

At the biological scale, many researchers have found microfragments and microfibers in several species of different trophic levels, such as zooplankton, fish, birds, arthropods, cetaceans, and pinnipeds [12]. Therefore, marine animals help us understand microplastics’ impact on the marine environment [13]. Regarding the consumption of microplastics, information is still scarce, and it is difficult to trace ingested particles in wild animals. Therefore, assessing the presence of microplastics in animal feces is one of the most widely used methods for tracking them [14]. As a result, microparticles have been obtained from several species and subsequently characterized to assess their potential impact on wildlife [15]. Experimentally, polystyrene-type microplastics have been shown to induce damage in chickens’ lung tissue by inducing apoptotic and autophagic mechanisms [16]. Identifying the type of plastic fragments (microparticles) circulating in the aquatic environment is relevant because it can relate to physiological alterations in animal species [17].

The extensive Chilean coastline and its significant hydrobiological resources (fish, mollusks, algae, among others) highlight the need to examine the presence of microplastics because of the potential harm to marine ecosystems, food safety, and public health. Studies in Chile have revealed the ingestion of microplastics by various marine species, including the anchovy (*Engraulis ringens*), the choro mussel (*Choromytilus chorus*), and king crabs (*Lithodes santolla*), with adverse impacts on growth and physiology [12,15,18,19]. A study in Las Cruces found microplastics in juvenile fish, suggesting color’s influence on consumption [9]. Another study identified microplastic particles in six fish species from the Biobío region, though an assessment of the broader impact on Chilean coastal wildlife remains lacking.

Consequently, all the information collected shows the presence of microplastics in the marine environment worldwide, including Chilean coasts, and it has been determined that animals can be affected by this contamination [20]. We hypothesized that birds entering the wildlife rehabilitation center may act as sentinels for environmental contamination caused by microplastics. Therefore, we aimed to evaluate microplastics in the feces of seabirds from the Biobío region that arrive at the Wildlife Rehabilitation Center (CEREFAS) of the Universidad San Sebastián.

## 2. Materials and Methods

### 2.1. Sample Collection

**Figure 1:**
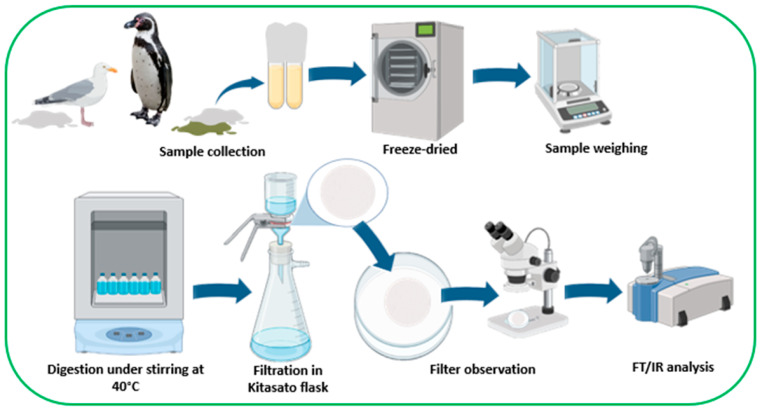
Methodological description of the study for the identification of microfibers in seabird feces. Figure created using Biorender (www.biorender.com, accessed on 24 November 2022).

Samples of bird droppings were collected from 13 gull chicks and 1 adult Dominican gull (*Larus dominicanus*), 8 Magellanic penguins (*Spheniscus magellanicus*), and 1 Humboldt penguin (*Spheniscus humboldti*) admitted to the Wildlife Rehabilitation Center of the Universidad San Sebastián between December 2021 and January 2022 (Figure 1). The gull chicks came from Tomé and Penco, the adult gull came from downtown Concepción, the Magellanic penguins came from Coronel and Tomé, and the Humboldt penguin came from Tomé.

During the general evaluation procedure of the birds upon their arrival at CEREFAS, fecal samples were collected. All fecal samples were collected prior to feeding the birds and during the first day of entry. For sample collection, the birds were housed in the hospitalization area of the aviary, which has a ceramic floor that was previously washed thoroughly to avoid contamination of the sample. Feces were collected using aluminum foil, deposited in glass test tubes previously washed with distilled water, and dried in an oven (Figure 1). In the case of the gull chicks, due to handling conditions to avoid stressing the birds, it was impossible to take individual samples, so a pool of feces was collected from the 13 animals. No birds were handled for the feces collection, and this procedure was under the supervision of the director of the rehabilitation center. After collection, the samples were labeled, transported to the Microplastics and Pollutants laboratory at Universidad San Sebastián, and stored in a −20 °C freezer. Sample analysis was carried out with the respective permits and collaboration agreements of the Universidad San Sebastián, the Agricultural and Livestock Service (SAG), and the National Direction of Fisheries–SERNAPESCA (exempt resolution No. 1125/2021).

### 2.2. Sample Treatment

The frozen samples were subjected to freeze-drying to eliminate all the water content. The freeze-dried samples were weighed and placed in a glass bottle, to which 300 mL of 15% KOH was added. The average weight of feces from penguins after freeze-drying was 1.6 g, adult gull feces weighed 0.2 g, and the gull chick pool weighed 1.7 g. These samples were then agitated at 100 rpm at a temperature of 40 °C until complete digestion, and every sample was transparent, homogeneous, and with sediments at the bottom. This process took from 3 to 12 days, depending on the sample. One Magellanic penguin sample could not be processed due to the fat content (steatorrhea), which hindered the digestion process and the precipitation of solids. Then, the samples were filtered using fiberglass filters in a Kitasato flask with the help of a vacuum pump. In order to filter the whole solution, each sample used a variable quantity of filters. A beaker with distilled water was used as a blank and placed next to the Kitasato flask during the time the sample was filtered. The distilled water was subsequently passed through a filter (Figure 1). This action aimed to identify if microparticles were circulating in the environment that could contaminate the filters. After each sample was filtered, the fiberglass filter was stored in a glass Petri dish and observed under a stereomicroscope to identify the plastic particles and their characteristics, such as shape and color.

### 2.3. Identification of the Microparticles

Fourier transform infrared (FTIR) spectroscopy is one of the most suitable, reliable, and commonly used methods for characterizing and identifying microplastics in samples [21,22]. Easily handled fibers and particles were analyzed to identify their composition. Fourier transform infrared spectroscopy (FTIR) [23] (Jasco–4600) was performed using a resolution of 4 cm^−1^, measuring in the range of 600–4000 cm^−1^. The obtained spectra were compared with the NICODOM IR Polymers and Additives Library containing 3954 spectra.

## 3. Results

**Figure 2:**
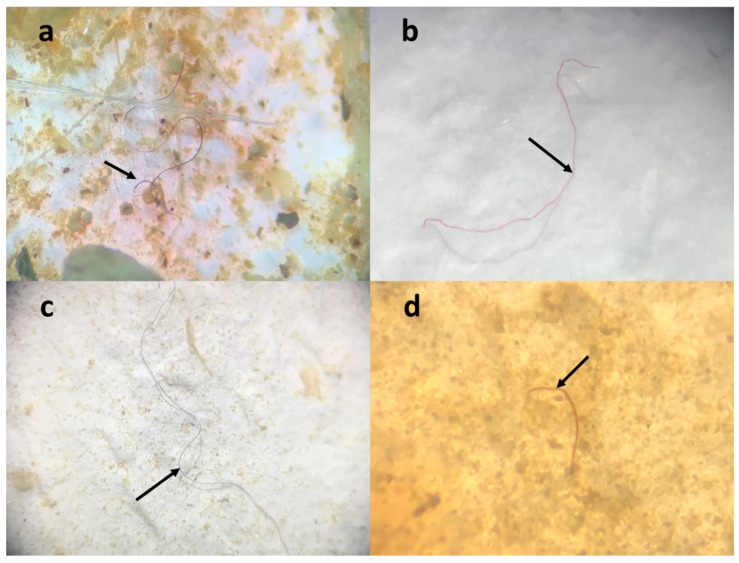
Microfibers identified in seabird feces (*Larus dominicanus, Spheniscus magellanicus*). (**a**) Blue microfiber identified in Magellanic penguin feces; (**b**) red microfiber identified in Magellanic penguin feces; (**c**) black microfiber identified in Dominican gull chick feces; (**d**) red microfiber identified in Dominican gull chick feces.

**Figure 3:**
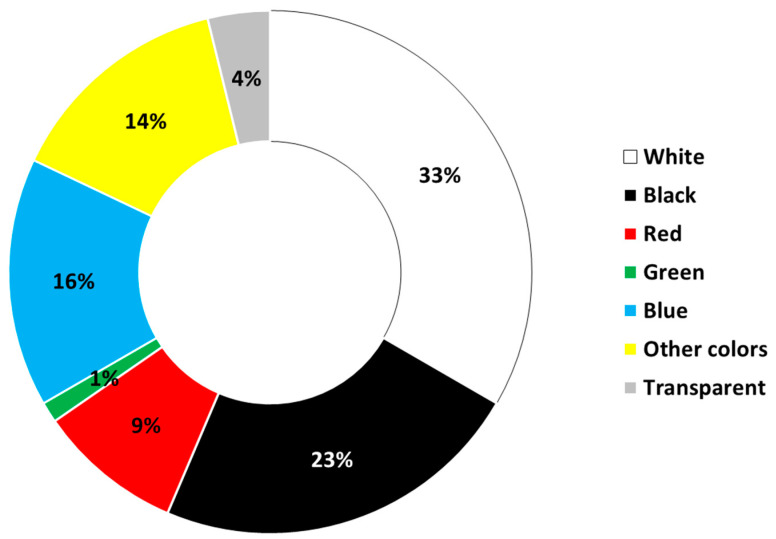
Fiber colors identified in Magellanic penguin and Dominican gull feces. Among the other colors, mainly yellow fibers were found.

Each bird’s total freeze-dried fecal material was used for the digestion process. The Magellanic penguins, chicks, and adult Dominican gulls’ feces contained fibers, while the Humboldt penguin sample did not. In total, 32 filters were observed; nine were controls (Table S1). Within these 32 filters, a total of 102 particles were identified, comprising 90 particles from the samples and 12 particles from the control group. After subtracting the particles found in the control filters, 78 particles were identified. In the 23 filters corresponding to the samples, 61% were positive for the presence of microfibers. The highest frequency of particles found corresponded to fibers (see Figure 2). The predominant color of the fibers was white (34%), followed by black (23%) (Figure 3).

**Figure 4:**
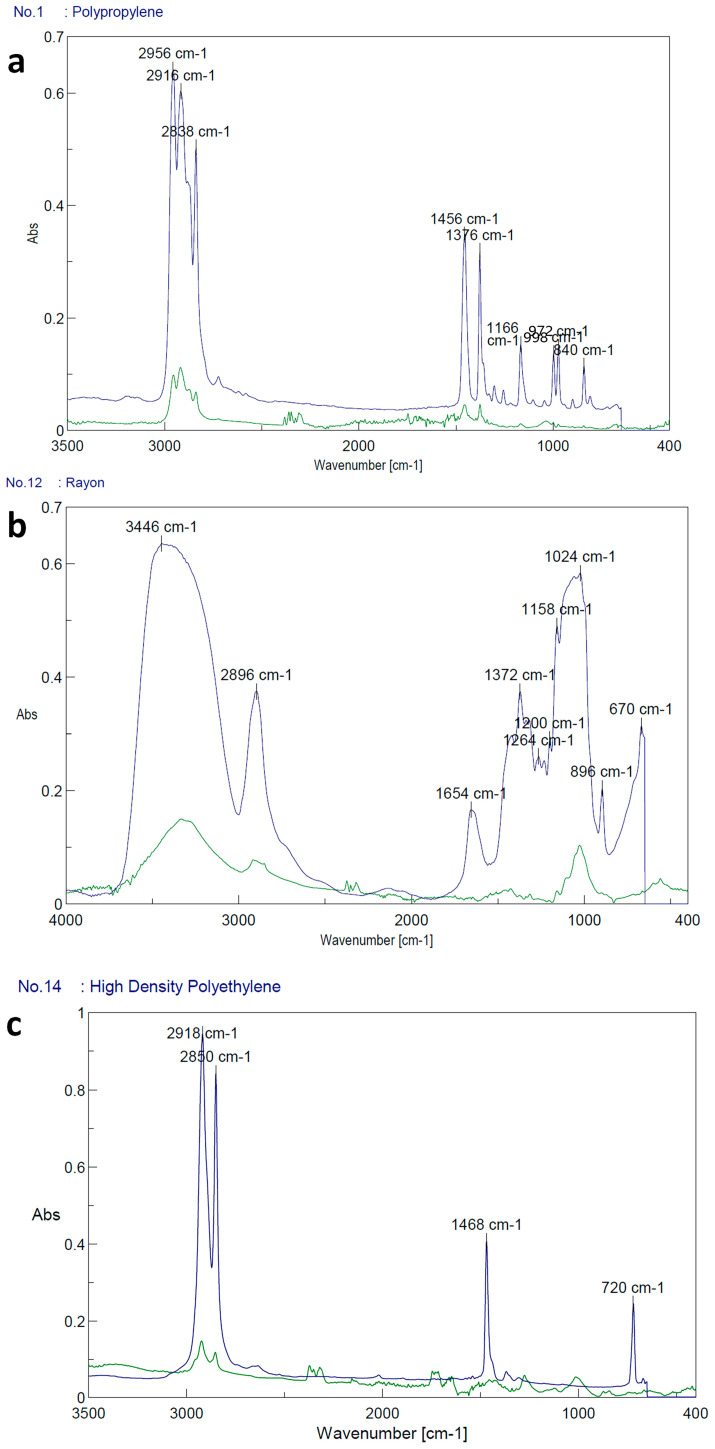
FTIR spectra. (**a**) According to the comparison with the database, the spectrum of the blue fiber from the gull chick feces pool had a composition of 83% polypropylene. (**b**) The spectrum of the cluster with a composition of 77% rayon found in gull chick feces. (**c**) The spectrum of fibers with a 53% high-density polyethylene composition is found in Magellanic penguin feces. The blue peaks correspond to the library, while the green peaks correspond to the sample.

Two larger fibers and pellets analyzed by FTIR presented polypropylene (83%) and rayon (77%) in their composition. The polypropylene fibers and the cluster belonged to the filters of the gull chick feces pool. The other fibers analyzed presented an organic composition (stearine acid, tristearin) or a polymer composition of less than 50% (high-density polyethylene in adult gull feces) (Figure 4).

## 4. Discussion

The present study identified the presence of plastic particles, mainly fibers, in the feces of two species of seabirds frequently found in the Wildlife Rehabilitation Center of the Universidad San Sebastián in the Biobío region. The presence of these particles in marine biota throughout Chile has been reported more frequently in recent years. Plastic microfibers have been reported in three species of pinnipeds with similar characteristics to those reported in the present study [13,14]. Likewise, species of economic importance in the fishing industry, such as the Chilean jack mackerel (*Trachurus murphyi*), sardine (*Strangomera bentincki*), and pejerrey (*Basilichthys australis*), have been reported as carriers of plastic microparticles [15]. On the other hand, in seabirds, these particles are increasing. Provencher et al. (2018) found that seabirds such as the northern fulmar (*Fulmarus glacialis*) excreted guano with plastic microparticles [24]. These researchers assessed the presence of microparticles in the feces and the stomachs of these birds and, as a result, concluded that the amount of microparticles present in guano was an indicator of the retention of microplastics in the digestive tract of birds [24]. These microparticles have been found even in remote locations such as Antarctica, where about 77% of the feces collected from king penguins (*Aptenodytes patagonicus*) had microfibers [25]. In the present study, all Magellanic penguin (*Spheniscus magellanicus*) feces evaluated contained plastic microfibers. This situation was expected, considering the distribution of this species in geographic areas with greater anthropogenic activity. Despite this, a recent study reported plastic debris in 97% of the birds studied on the coasts of southern Argentina [26]. This penguin species has a wider distribution than the Humboldt penguin and is capable of seasonal migration throughout the southern zone of South America [27].

The present study also reported the presence of plastic microfibers in the feces of chicks and an adult Dominican gull (*Larus dominicanus*). Regarding the Dominican gull, the results of previous studies on the ingestion of plastic material are very variable. Yorio et al. (2022) found that the prevalence of plastic debris was relatively low in Dominican gull nests in northern Argentina, while Lenzi et al. (2016) found plastic debris in one out of four gull regurgitations off the coast of Uruguay [28,29]. In addition, chick gulls could be influenced by the reduced period of feeding, which influences the likely amount of microfibers that can be ingested. The present study cannot provide a clear conclusion on the presence or absence of plastic particles in adult Humboldt penguins; therefore, we suggest increasing the sample size for future investigations.

The plastic particles found were predominantly fibers. Pellet-shaped particles and others without a defined shape (clumps) were found in smaller proportions. This could suggest a large concentration of these microfibers in the marine environment, the origin of which may be difficult to identify. However, it is known that the textile industry is one of the most polluting activities worldwide and could also be a source of these particles [30].

Regarding the characteristics of the particles identified in the present work, there was a clear predominance of white at 34% and black at 23%. These results are in partial agreement with several similar studies. For example, in the findings of Pérez-Venegas et al. (2018), higher amounts of blue and white plastic fibers were identified in otariid feces from the Chilean and Peruvian coasts [14]. However, in guano from northern fulmar, a seabird, the predominant fibers were blue, followed by black and, in smaller quantities, red fibers [31]. In fish, which are the main food of many seabird species, the predominant color of fibers is blue, followed by black and, in smaller quantities, red fibers [9]. In the Biobío region, it has been documented that microfibers found in fish samples are mainly composed of red microfibers [15]. However, in the present study, blue accounted for only 16% of the identified fibers.

This study focused only on species that entered CEREFAS due to other potential threats. Therefore, although the sample was small, the conditions found in the birds that entered this center generally reflect the population’s health and the ecosystem’s conditions. These seabirds that entered CEREFAS came from the coasts of the Biobío region, in which Talcahuano is registered as the bay in Chile with the third highest contamination by plastic material [1].

In the case of the Dominican gull (*Larus dominicanus*), this species is characterized by its high flexibility and adaptability to urban environments, being able to feed on food waste by scavenging through garbage [32]. For this reason, it would be expected to find many particles and plastic microfibers. However, during the study, the number of adult gulls decreased, and more chicks were recorded, in which a low presence of plastic microfibers was found. This may be because they present a more selective feeding habit at this stage, and they may be less exposed due to their limited movement and dependence on their parents [33]. For this reason, it is still necessary to conduct a study at a time when it may be feasible to sample a larger number of adult gulls.

Regarding the spectra obtained from the particles that FTIR could analyze, it was identified that they corresponded to materials such as polypropylene (PP), rayon, and high-density polyethylene (HDPE). PP and HDPT are widely used but mainly in plastic products (containers, rigid structures). At the same time, rayon is a semi-synthetic fiber made from natural sources of regenerated cellulose, such as wood and related agricultural products, used with textiles. The circulation of HDPE on the coasts and in commercial fish in central Chile has been reported previously [15,34]. Although polypropylene (PP) and rayon were reported here for the first time in the feces of the birds studied in this area, particles of this composition have been identified in the digestive tract of birds in different regions of the world, such as the United States and China [35,36].

## Data Availability

The data presented in this study are available in supplementary material.

## Supplementary Materials

The following supporting information can be downloaded at https://www.mdpi.com/article/10.3390/ani13182840/s1. Table S1: Characterization of the particles.
